# Supplementary material for: Quantitative MRI biomarker for classification of clinically significant prostate cancer: Calibration for reproducibility across echo times
Source: J Appl Clin Med Phys. 2024 Oct 7;25(11):e14514. doi: 10.1002/acm2.14514 (PMC11539966; doi:10.1002/acm2.14514)
Supplement: Supplementary file 1 — Supporting Information [file ACM2-25-e14514-s001.pdf]

Supplemental Materials

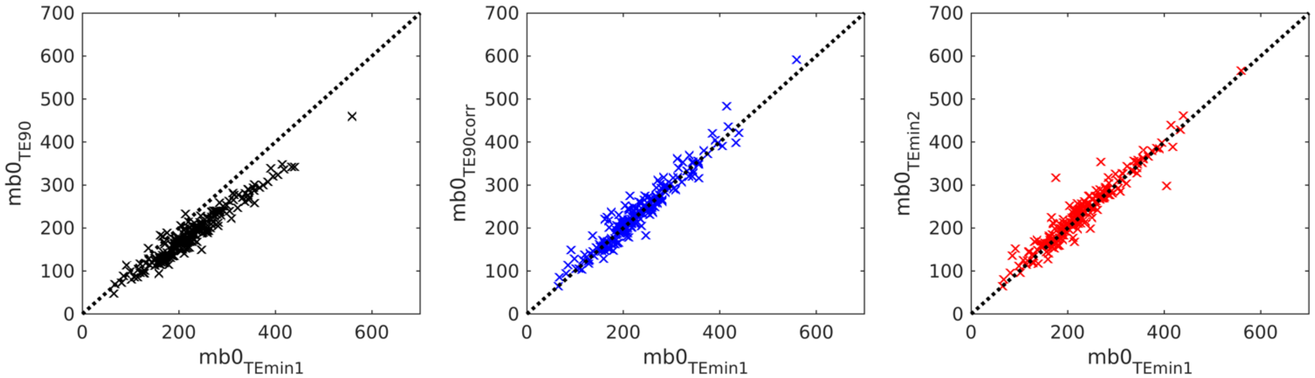

Supplementary Figure 1: Comparison of *mb0* $TEmin_1$ for all cases to *mb0* TE90, *mb0* TE90corr and *mb0* $TEmin_2$. Black dashed lines indicate hypothetical perfect relation.

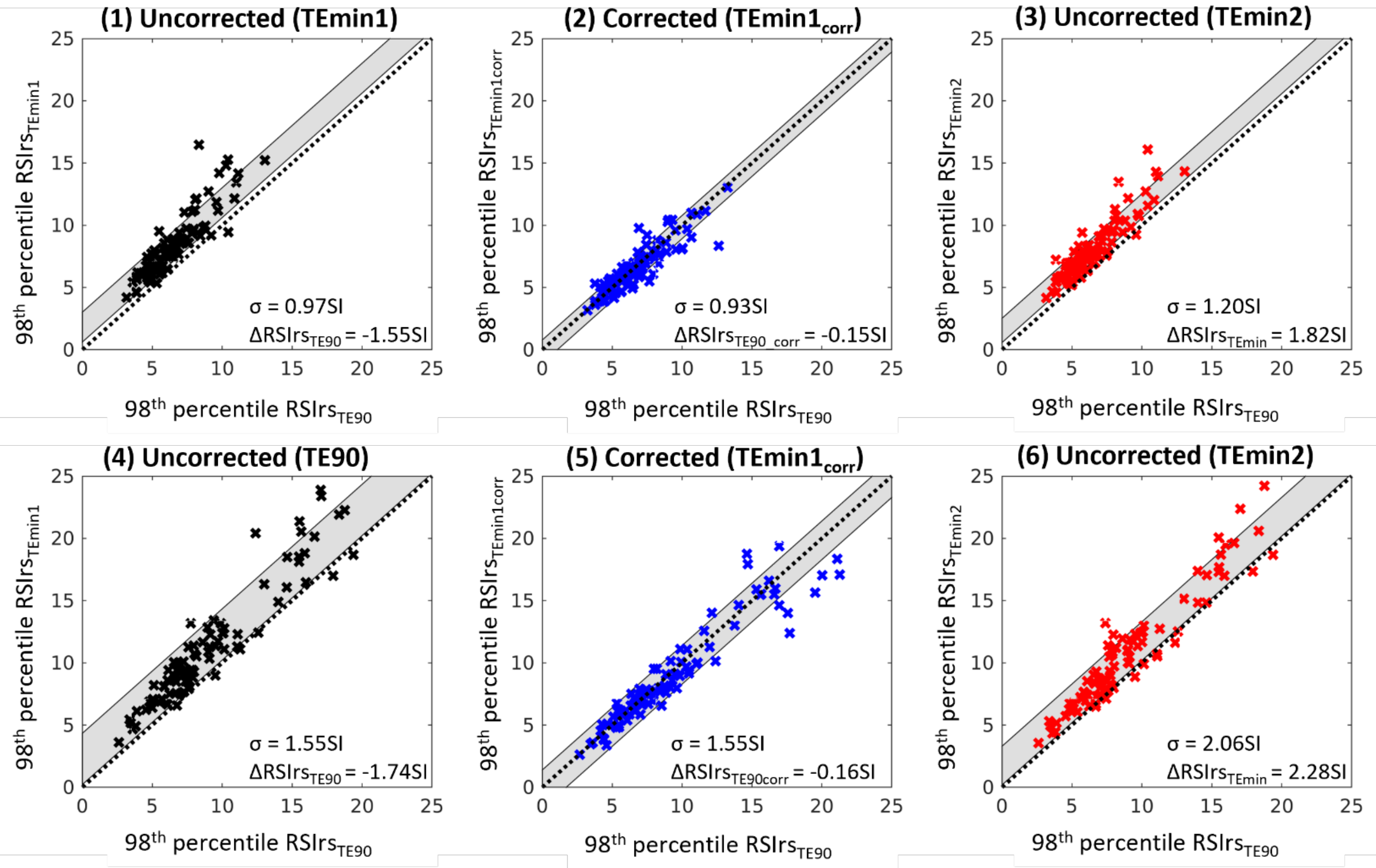

Supplementary Figure 2: *comparison of the 98th percentile of RSIrs$_{TE90}$ for benign cases (1-3) and csPCa (4-6) cases within the prostate to the 98th percentile of RSIrs$_{TEmin1}$ (1,4), RSIrs$_{TE90corr}$ (2,5) and RSIrs$_{TEmin2}$ (3,6). Standard deviation (indicated by σ as well as gray color wash) and mean difference of the reference, 98th percentile of RSIrs$_{TE90}$, to the 98th percentile of RSIrs$_{TEmin1}$, RSIrs$_{TEmin2}$ and RSIrs$_{TE90corr}$ (ΔRSIrs) indicating model bias. Black dashed lines indicate hypothetical perfect relation.*

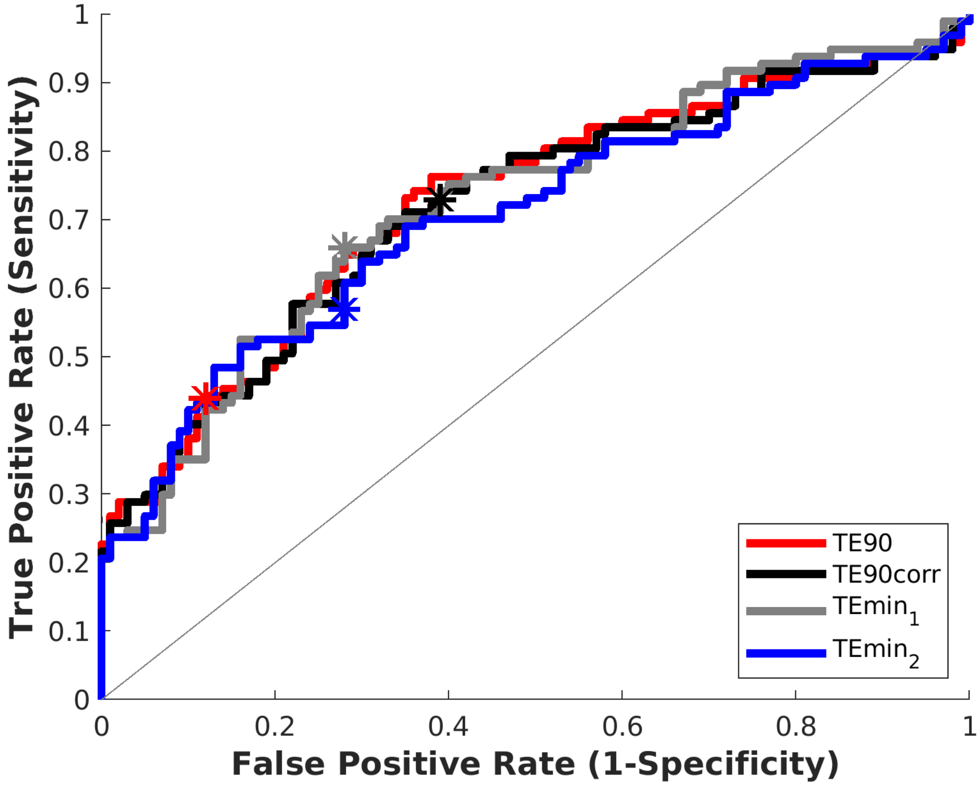

Supplementary Figure 3: ROC curve for classification of csPCa using the 98th percentile of RSIrs within the prostate as the biomarker. The grey line indicates a model performance better than randomness. Crosses indicate the classification performance at the threshold defined by Youden index.

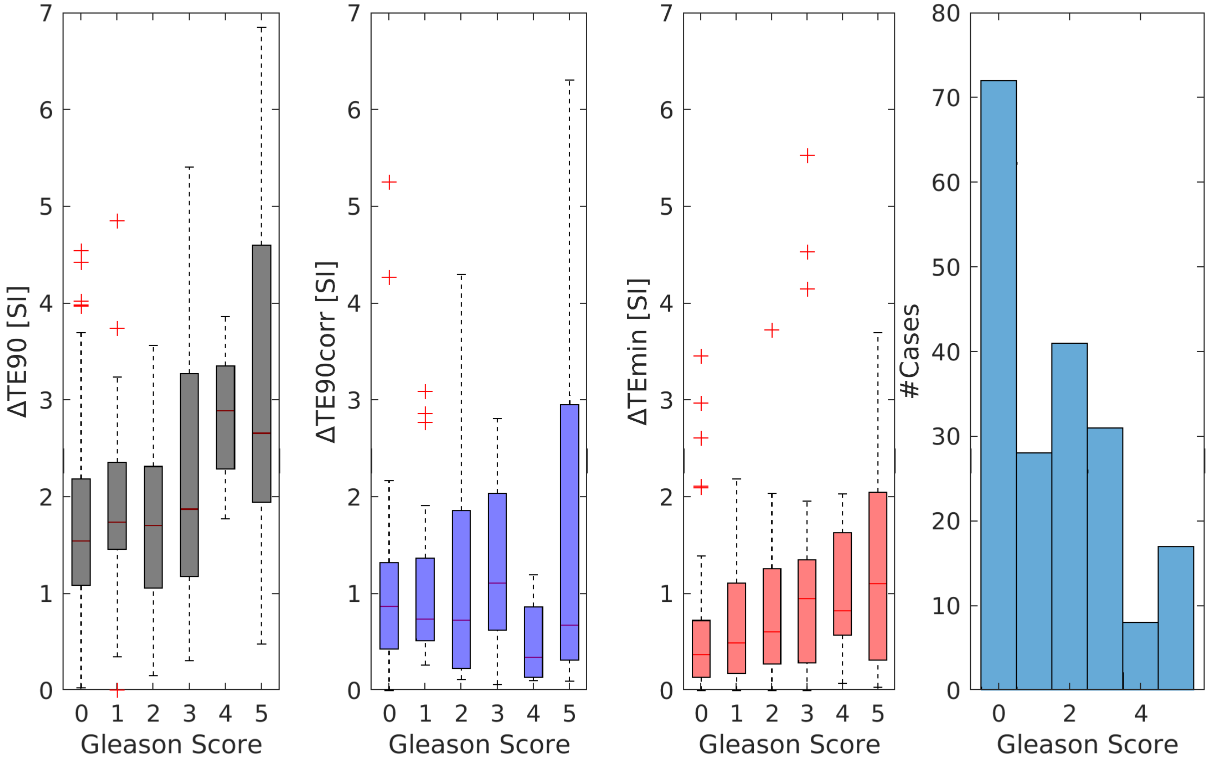

Supplementary Figure 4: *Absolute differences between TE90, TE90corr, and $TEmin_2$ and the reference acquisition $TEmin_1$ for each Gleason grade group. Gleason Score = 0 indicates benign cases. The histogram illustrates the frequency distributions of Gleason scores in the patient cohort.*

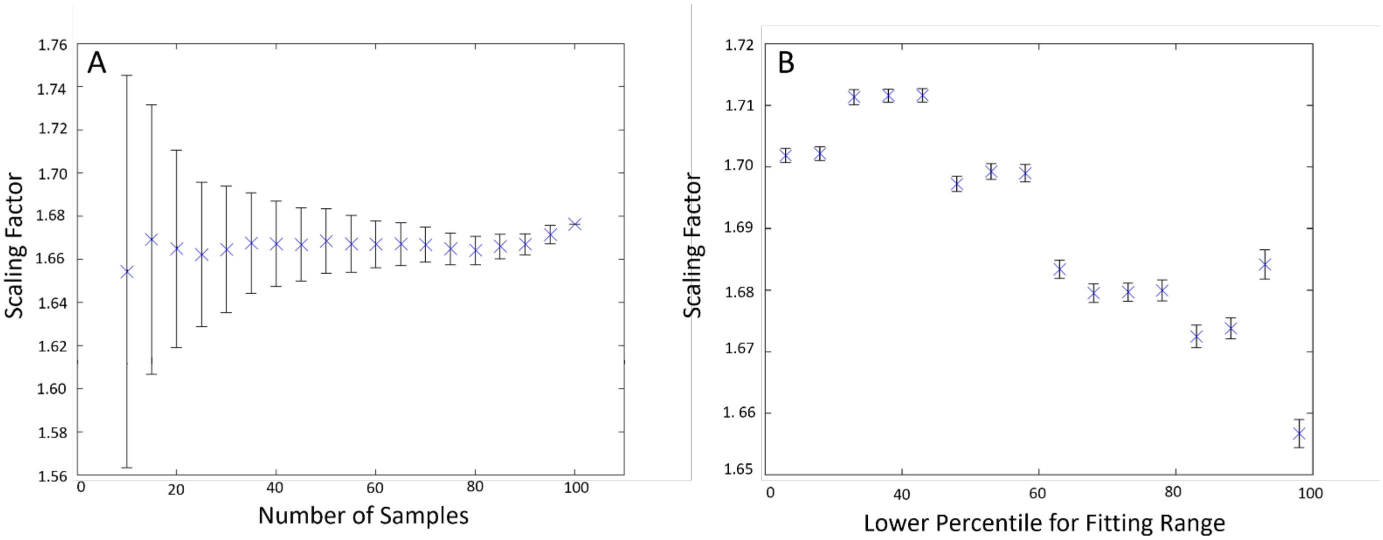

Supplementary Figure 5: *Scaling factor analysis for C1 RSI map. A) Mean scaling factor and 95% confidence interval derived from 1000 bootstrap samples for varying sample sizes (N). For N≥35, the 95% confidence interval lies completely within 2% of the scaling factor estimate for the full dataset. B) Mean scaling factor and 95% confidence interval across different fitting ranges. E.g., the data point at 99th percentile indicates the scaling factor estimated for voxels in the top 1 percent of signal within the prostate of each patient. Likewise, the data point at 60th percentile represents the scaling factor for voxels in the top 40 percent of signal within the prostate of each patient. For fitting ranges using the 60th percentile or greater as the minimum threshold, the scaling factor point estimate and 95% confidence interval are within 2% of the scaling factor estimate presented in the main text (using 95th – 99th percentiles as the fitting range). Lower ranges for fitting yield estimates that are approximately 3% greater than the scaling factor presented in the main text.*
